# Primary Pulmonary Epithelioid Angiosarcoma: A Rare Case Presentation of Bilateral Pneumothoraces

**DOI:** 10.7759/cureus.6514

**Published:** 2019-12-30

**Authors:** Saif Faiek, Hafsa Tariq, Deepti Upparapalli, Aditya Bansal, Sreeja Sompalli

**Affiliations:** 1 Internal Medicine, AtlantiCare Regional Medical Center, Atlantic City, USA; 2 Critical Care Medicine, AtlantiCare Regional Medical Center, Atlantic City, USA

**Keywords:** epithelioid angiosarcoma, pulmonary neoplasm, lung cancer, bilateral pneumothoraces

## Abstract

Angiosarcoma is a rare malignant neoplasm with a very poor prognosis that originates from the vascular endothelium and accounts for only 1%-2% of all sarcomatous malignancies. It is most commonly present in the deep soft tissues. Still, a wide range of primary sites, including the adrenals, thyroid, skin, and bone, are encountered. Here, we report a 52-year-old female with a past medical history of hypertension who presented with chest pain. Her chest images showed bilateral pneumothoraces with diffuse cystic lung disease. She underwent bilateral video-assisted thoracoscopy with a tissue biopsy, which was consistent with epithelioid angiosarcoma.

## Introduction

Angiosarcoma is a rare malignant neoplasm with a very poor prognosis that originates from the vascular endothelium and accounts for only 1%-2% of all sarcomatous malignancies [1]. It arises most often in the deep soft tissues, although other primary sites have been reported, including the adrenals, thyroid, skin, and bone [2]. Primary pulmonary angiosarcoma is an extremely rare malignancy, with only a few cases reported in the English literature. Its rarity and the consequent low index of suspicion makes the clinical diagnosis challenging [3]. Here, we report a case of primary angiosarcoma of the lung with reviewing the relevant literature.

## Case presentation

**Figure 1:**
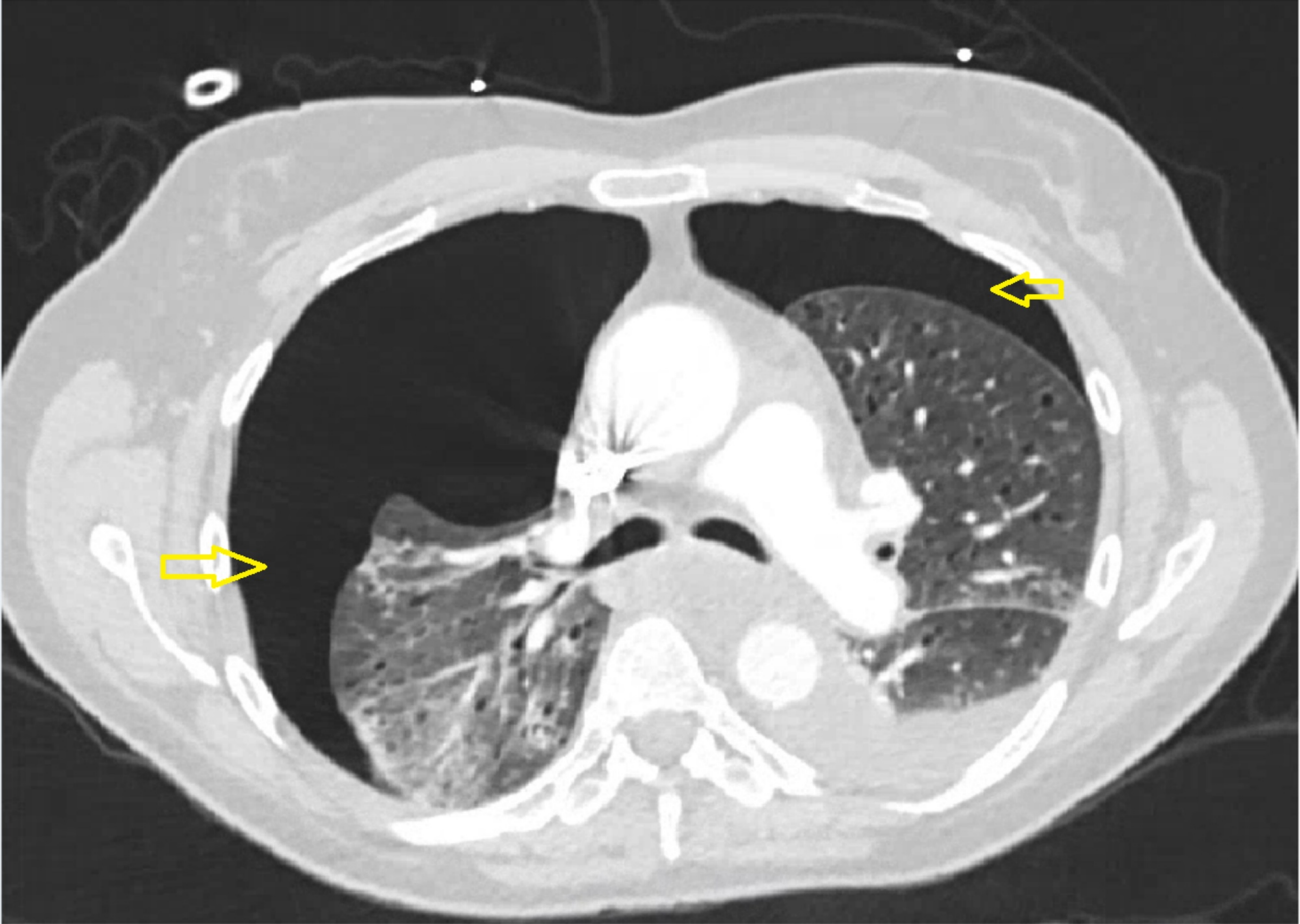
CT angiography of the chest showing bilateral pneumothoraces (yellow arrowhead); large right pneumothorax and moderate left pneumothorax with a left pleural effusion and diffuse bilateral cystic lung disease.

A 52-year-old Caucasian woman with a past medical history significant for hypertension and status post negative cardiac catheterization for chest pain presented with progressively worsening chest pain for one month. The pain was mid-sternal, dull in character, intermittent, radiating to the back, exacerbated with coughing, deep breathing, and exertion. On examination, she was tachycardic, tachypneic, and hypoxic. CT angiography of the chest, as part of the initial workup, showed bilateral pneumothoraces with bibasilar atelectasis and a left-sided pleural effusion. Multiple significant bilateral cystic lesions were noted in the lungs (Figure 1). 

**Figure 2:**
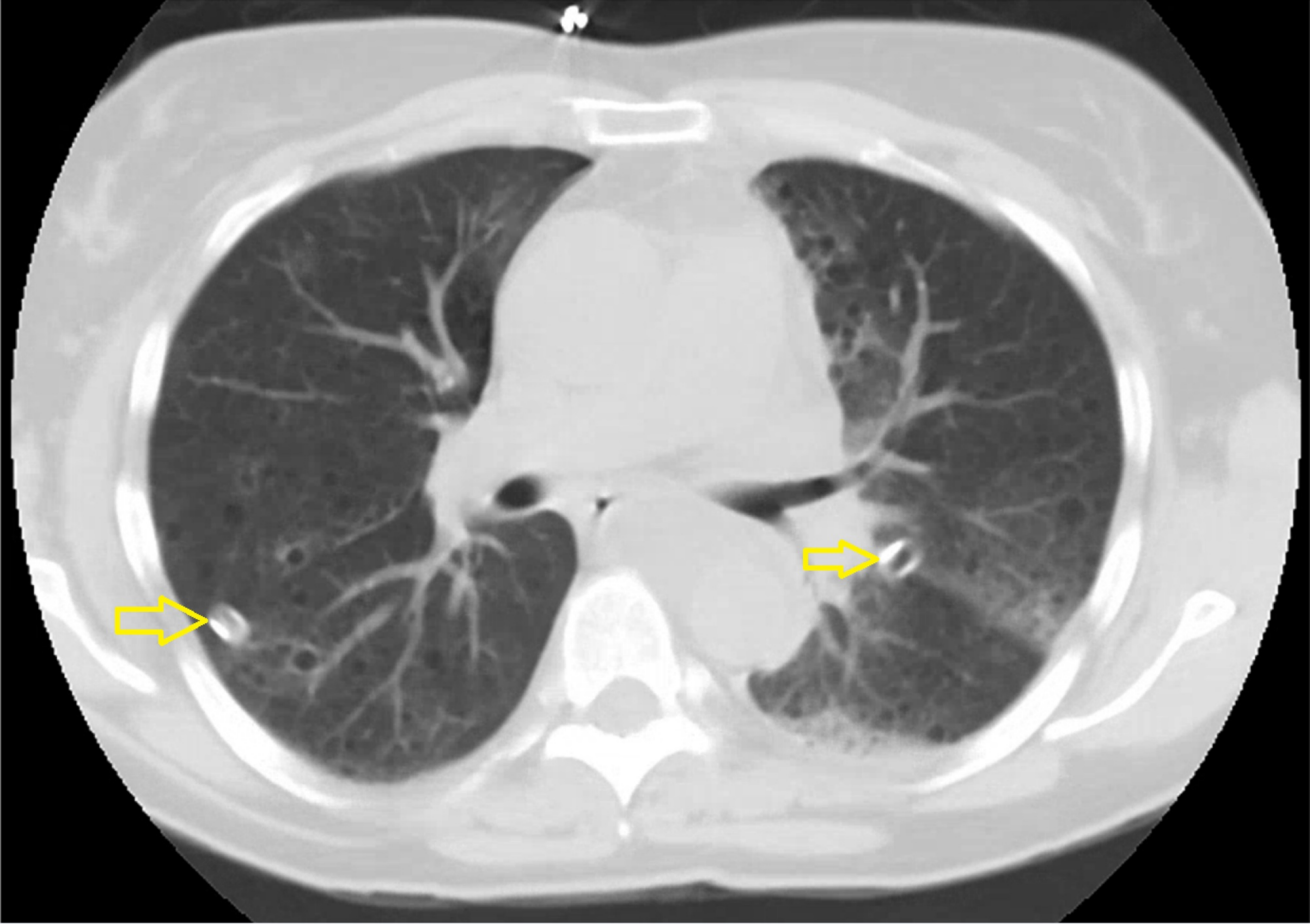
CT chest without contrast showing bilateral chest tubes (yellow arrowhead) with a near-complete resolution of bilateral pneumothoraces.

Emergent bilateral chest tubes were placed with a resolution of the pneumothoraces in the chest x-ray and CT chest without contrast following insertion (Figure 2). Repeat CT chest showed an anterior mediastinal opacity/mass with a right paratracheal lymph node along with multiple thin and thick-walled cysts throughout bilateral lung fields. She also had bilateral adrenal angiomyolipoma, which was unchanged from the prior scan. There was also a right anterior renal mass. 

**Figure 3:**
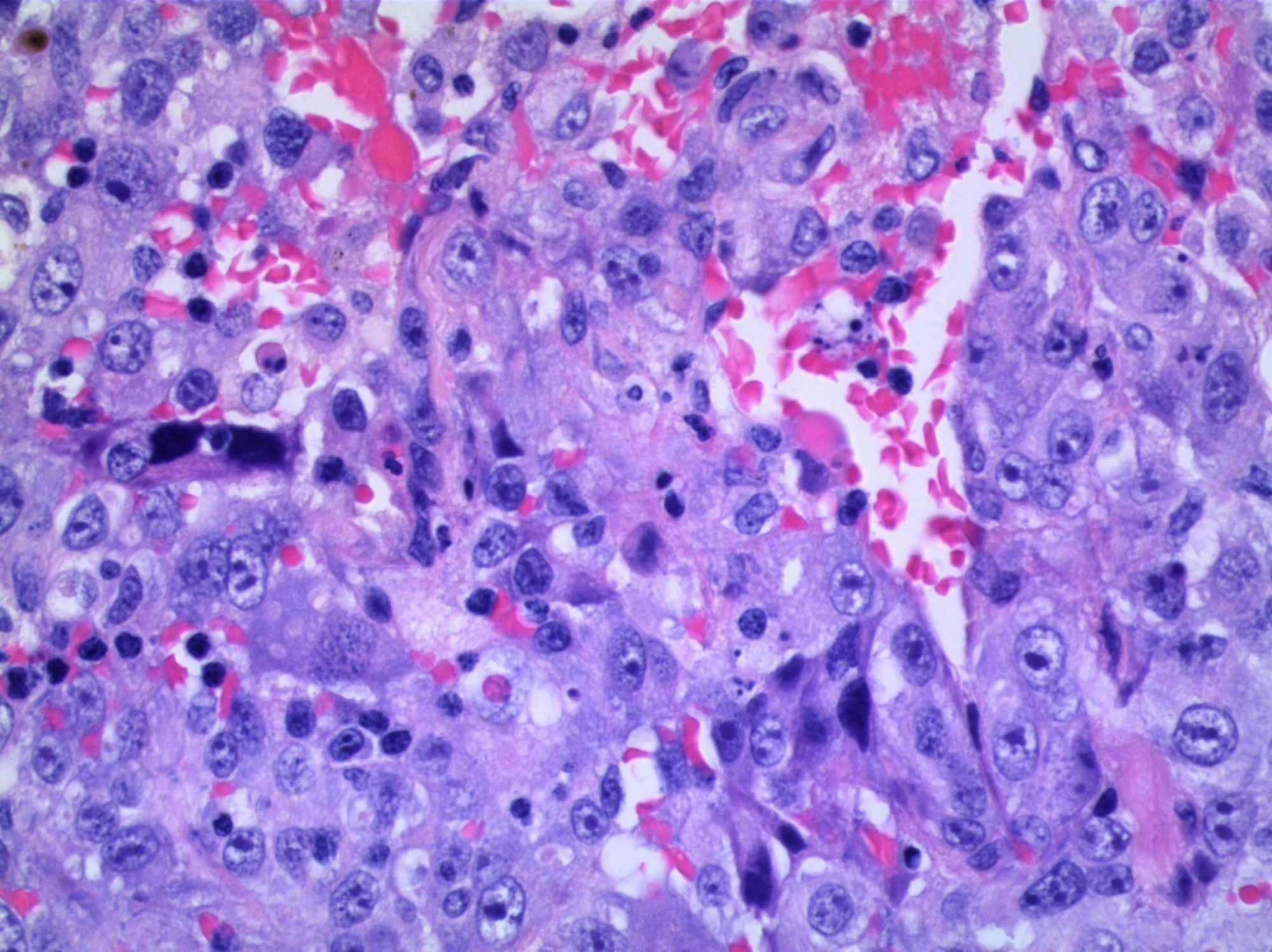
High-power image of hematoxylin and eosin stain showing highly malignant appearing cells with vesicular nuclei, prominent nucleoli, and moderately abundant eosinophilic to amphophilic cytoplasm.

**Figure 4:**
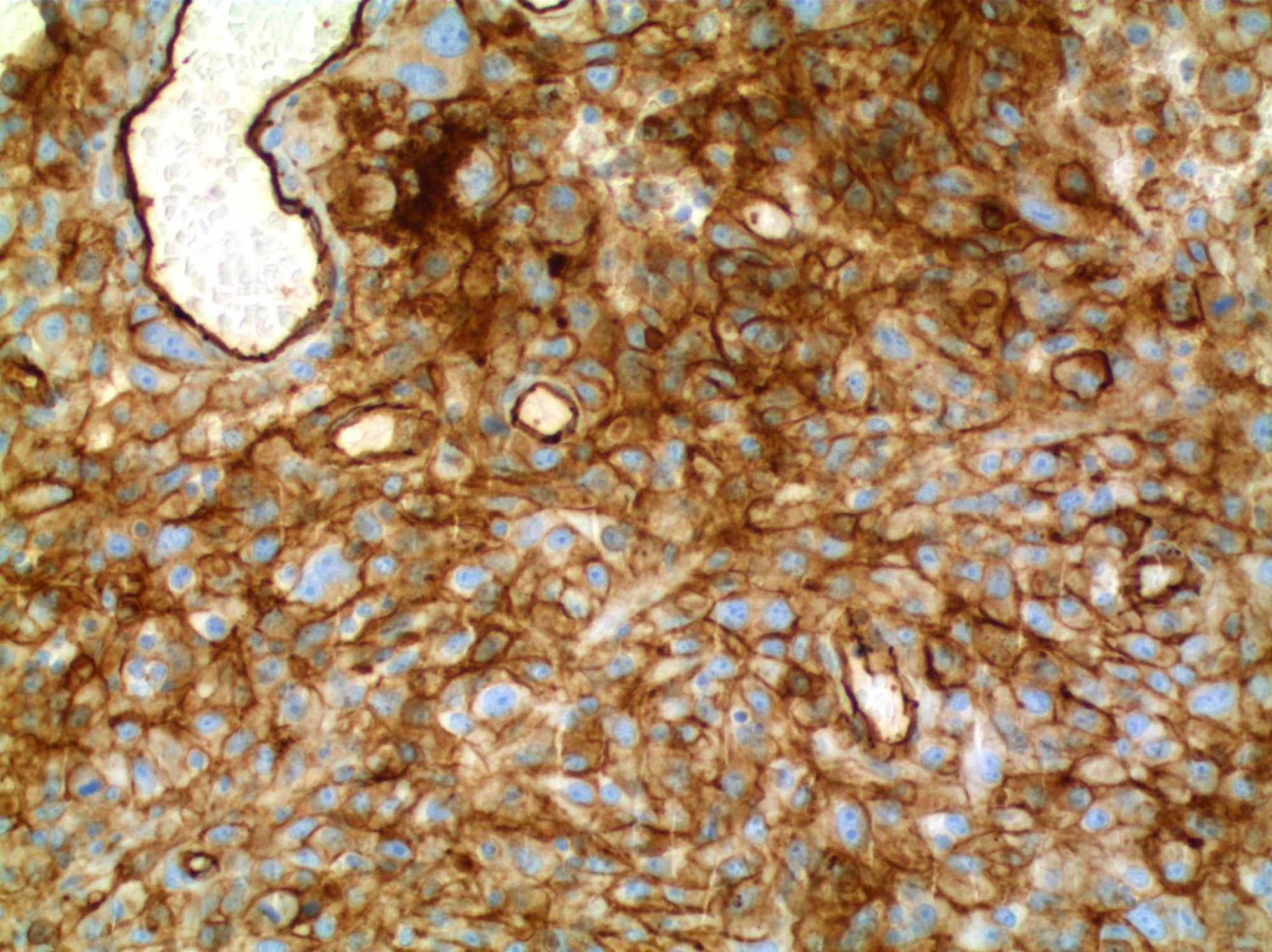
CD31 stain showing strong membranous staining over the tumor cells, indicating endothelial origin with all other immunohistochemistry stains done were negative and very weak.

Given the mentioned findings, there was a concern for a more diffuse or systemic disease process. Hence, she underwent an extensive workup of the diffuse cystic pulmonary lesions. Pleural fluid studies were consistent with exudative etiology; however, cultures were negative. HIV screen and autoimmune workup were negative. Bilateral video-assisted thoracoscopy with right middle and lower lobe wedge resections were done for a more definitive diagnosis. She also underwent bilateral talc pleurodesis as there was a concern for the recurrence of pneumothorax. Tissue pathology was consistent with poorly differentiated malignant neoplasm. Immunohistochemical stains were positive for CD31, and negative for CD34 and factor VIIIRA. The morphology and CD31 expression was highly suggestive of epithelioid angiosarcoma (Figures 3, 4).

The patient was offered complete staging and further workup for her diagnosis, but she declined any further evaluation and died within a month of diagnosis. On the basis of the above radiological and pathological findings, and in the absent evidence of any other primary source, the tumor was classified as primary epithelioid angiosarcoma of the lung.

## Discussion

Epithelioid angiosarcoma is a rare malignant vascular neoplasm of the lung, which approximately accounts for 0.001% of all cancers. It is associated with a poor prognosis with an average survival of nine months [3]. The mean age of the reported cases was 54.7 years [4]. Etiology remains unknown with possible predisposing factors including polyvinyl chloride and thorium dioxide. There is no clear-cut consensus on presentation or imaging between primary and metastatic pulmonary epithelioid angiosarcoma. The presentation can be similar to symptoms found in all lung cancers such as dyspnea, chest pain, hemoptysis, cough, and weight loss. The most common radiograph finding is multiple nodules, although linear infiltrates, pleural effusion, and diffuse alveolar infiltrates can be seen. Although a thin-walled cavitary lesion is usually not considered to be a malignancy, angiosarcomas seem to an exception to this rule [5]. Primary pulmonary angiosarcoma showing multiple infiltration or consolidation suggests a poor prognostic factor [1]. Definitive diagnosis is made by histopathological and immunohistochemical findings. Treatment options include surgery, chemotherapy, and radiation. Surgery is better for loculated lesions. Vascular embolization may be tried to reduce the size of the tumor. The study done by Wilson et al. showed a complete radiographic response of disease by a combination of gemcitabine and taxotere [6]. However, Ozcelik et al. mentioned prognosis of pulmonary angiosarcoma has been poor, with almost all patients die within months of initial presentation [7]. Hence, accurate diagnosis and timely treatment might benefit these patients.

## Conclusions

Diffuse cystic lung diseases are a group of diverse disorders characterized by multiple thin-walled air-filled spaces within the lung parenchyma. Rarely, cystic lung lesions develop as the result of extensive underlying neoplastic processes (both primary and secondary), such as sarcomas and mesenchymal tumors. One such malignancy is epithelioid angiosarcoma, which is extremely aggressive, rare, and can often present as diffuse cystic lung disease. To make matters worse, this is often complicated with the presence of pneumothoraces, which infers a very poor prognosis, such as in the patient we encountered.
